# Investigation of the Carbon Monoxide Gas Sensing Characteristics of Tin Oxide Mixed Cerium Oxide Thin Films

**DOI:** 10.3390/s120302598

**Published:** 2012-02-27

**Authors:** Sardar M. A. Durrani, Mohammad F. Al-Kuhaili, Imran A. Bakhtiari, Muhammad B. Haider

**Affiliations:** Physics Department, King Fahd University of Petroleum and Minerals, Dhahran 31261, Saudi Arabia; E-Mails: kuhaili@kfupm.edu.sa (M.F.A.-K.); iab@kfupm.edu.sa (I.A.B.); mhaider@kfupm.edu.sa (M.B.H.)

**Keywords:** thin film, AFM, XPS, CeO_2_, SnO_2_, CO sensor, semiconductor gas sensor

## Abstract

Thin films of tin oxide mixed cerium oxide were grown on unheated substrates by physical vapor deposition. The films were annealed in air at 500 °C for two hours, and were characterized using X-ray photoelectron spectroscopy, atomic force microscopy and optical spectrophotometry. X-ray photoelectron spectroscopy and atomic force microscopy results reveal that the films were highly porous and porosity of our films was found to be in the range of 11.6–21.7%. The films were investigated for the detection of carbon monoxide, and were found to be highly sensitive. We found that 430 °C was the optimum operating temperature for sensing CO gas at concentrations as low as 5 ppm. Our sensors exhibited fast response and recovery times of 26 s and 30 s, respectively.

## 1. Introduction

There is an ever-increasing demand for gas sensors in various fields. Particular attention has been devoted to the monitoring of carbon monoxide (CO). More precise control of the air/fuel ratio in a combustion processes can yield significant gains in efficiency and result in substantial savings in fuel consumption. The flue gas concentration of CO is a reliable and accurate indication of the completeness of combustion, as it is the most sensitive indicator of unburned combustible losses. Metal oxide semiconductors have been employed in the detection of CO. One of the advantages of these materials is that they enable high temperature operation, making them unique for hostile industrial applications. Moreover, many gas reactions are plausible only at such elevated temperatures. The basic property of metal oxides that is of interest in gas-sensing applications is the dependence of their electrical conductivity on the ambient gas. Most metal oxide semiconductors are naturally of n-type conductivity due to the presence of a large number of oxygen vacancies. When such a material is exposed to the atmosphere, oxygen molecules are chemisorbed to the grain boundaries and pick up electrons from the conduction band and create a space charge layer between the grains [1]. This leads to the formation of Schottky barriers at the surfaces of the grains, and increases the resistivity of the material [2]. Exposure of the material to reducing gases (such as carbon monoxide) causes a reaction of these gases with the chemisorbed oxygen, increasing the electronic conduction and reducing the resistance [1,2]. The sensing properties are based on surface reactions and are greatly affected by the microstructure of the material [3].Thin films offer the added advantage of higher surface-to-volume ratio. In addition to the choice of the semiconducting oxide, other film parameters that are widely known to affect the sensing properties of a thin film are surface roughness, stoichiometry, and porosity, basicity. Most of the commercially available gas sensors are based on thick-film metal oxide materials deposited on ceramic heater substrates [4]. A common shortcoming of such thick-film devices is their high level of heating power consumption. This level can be reduced by about one-order of magnitude using micro-machined heater substrates [4]; in addition their response and recovery times are much longer compared to the thin film sensors. The established state-of-the-art in silicon micromachining is still the use of evaporated or sputtered thin-film metal oxide materials [4].

Several metal oxides have been tested as CO gas sensors. Tin oxide based gas sensors are among the most widely used semiconductors for detecting CO [5–9]. More recently pure CeO_2_ thin films have also been investigated for their application as CO gas sensors [10], and were found to be highly sensitive to CO. These high sensitivities were attributed mainly to the film porosity. CeO_2_ films are known to be highly porous [3,11]. It is known that additions of various second-phase oxides and/or catalysts (such as Pd, Pt, and CuO *etc.*) improve sensing characteristics for CO detections [5–7]. Motivated by the high sensitivity of SnO_2_ thin films and the high porosity of CeO_2_ thin films, we prepared SnO_2_ mixed CeO_2_ thin films. Here we report the significant improvement in term of response time and detection limit observed for these mixed films.

## 2. Experimental Section

**Figure 1.**
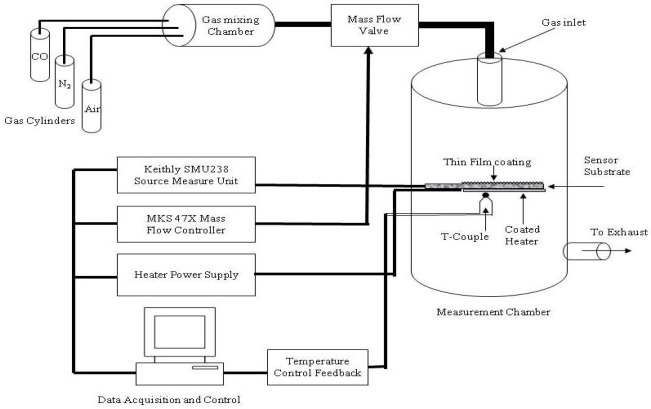
Experimental setup for sensitivity measurements.

Thin films of SnO_2_ mixed CeO_2_ were prepared by co-evaporation using physical vapor deposition. For this purpose CeO_2_ was evaporated by e-beam while SnO_2_ was evaporated by thermal evaporation simultaneously. Ratios of the two materials were controlled by quartz crystal monitor. The evaporation rates of CeO_2_ and SnO_2_ were fixed at 3.2 Ǻ/s and 0.8 Ǻ/s respectively (75% CeO_2_ and 25% SnO_2_), with the total rate of 4 Ǻ/s. The films were prepared in a Leybold L560 box coater pumped by a turbomolecular pump. The system was initially pumped to a base pressure of 1 × 10^−4^ Pa. Before deposition, the materials were slowly outgassed, with a shutter blocking the vapors from reaching the substrate. The films were deposited on unheated substrates. The substrates were rotating during the deposition, and the source-to-substrate distance was 40 cm. The evaporation rates and thickness of the films were controlled by a quartz crystal thickness monitor. For different purposes of film characterization, the films were simultaneously deposited on tantalum substrates (for X-ray photoelectron spectroscopy, XPS), fused silica substrates (for optical measurements), and alumina substrates (for gas sensing measurements). After the films were deposited, they were removed from the coating chamber and exposed to the ambient atmosphere. Subsequently, all samples were annealed in air at 500 °C for two hours in order to thermally stabilize the films prior to sensing measurements. XPS was performed using a VG Scientific MKII spectrometer with an Al *K*α (1,486.6 eV) X-ray source. The instrumental resolution was 1.2 eV, with a slit width of 0.6 cm. Prior to the XPS analysis; the samples were transferred in air to the XPS analysis chamber. The C 1s peak of hydrocarbon contamination, at a binding energy of 284.5 eV, was used as an energy reference. During the XPS analysis, the samples were maintained at ambient temperature at a pressure of 5 × 10^−7^ Pa. Normal-incidence transmittance and reflectance, over the wavelength range 300–1,200 nm, were measured using a Jasco V-570 double beam spectrophotometer. The thicknesses of the annealed films were measured using a surface profilometer (AMBIOS XP-2), and was found to be 220 nm. The AFM images of the sample were acquired using tapping mode of Digital Instrument’s (VEECO) Innova SPM and Nano-Drive Controller system. For these images the Phosphorus (n) doped Silicon probes were used. These probes have the nominal tip radius of less than 10 nm, tip height of 15 to 20 μm and the resonant frequency of about 300 kHz. The AFM images were processed using Digital Instruments SPMLab Version 7.0 software for leveling and noise removal. The gas-sensing measurements were made on the films deposited on top of alumina substrates with platinum interdigitated electrodes (for electrical measurements). A platinum heater was printed on the backside of the substrate. The gas-sensing measurements were carried out in the environmental test chamber shown in Figure 1.

The desired CO concentration in ppm was achieved using a stainless steel gas mixing chamber in combination with MKS 647 controller and the 100SCCM mass flow controller. A Leybold model combiVAC 2T digital pressure measurement unit for low pressure and a 200-PSI high pressure gauge from USG was used to measure the chamber pressures. The chamber was first vacuumed to the pressure level of ∼1 × 10^−7^ mbar using Leybold Model PT 50 pumping system which is composed of a roughing and turbo molecular pumps. Initially a known volume of High Purity CO gas was added to the mixing chamber using the 100 SCCM Mass flow controller and then it was mixed with the required volume of air as buffer gas to achieve the desired CO concentration. The concentration of CO was varied between 5–5,000 ppm. The volume of gas to be injected was controlled by the duration (dwell time) of valve opening and its flow rate. The temperature of the sensor was controlled with a programmable BK Precision power supply (BK1770), K-type thermocouple (feedback element) and a PC PID temperature controller. The sensor temperature was varied in the range 300–600 °C. The sensor current (under a given bias voltage) was measured using a source measure unit (Keithly 238). The resistance of the films was calculated as the ratio between the applied bias voltage and the measured current. A personal computer was used to control all the operations (gas injection, temperature measurement and control, and current measurement) using LABVIEW via a GPIB interface.

## 3. Results

### 3.1. Chemical Analysis (XPS)

**Figure 2.**
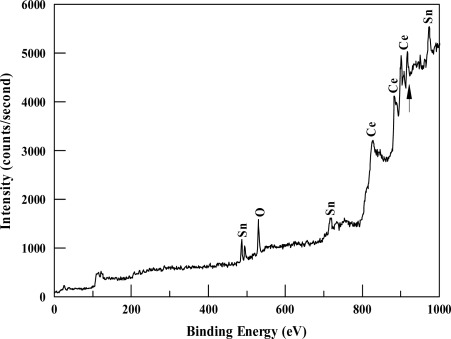
XPS survey spectrum of the deposited films.

The chemical state of the films was investigated using XPS. Figure 2 shows an XPS survey scan of a typical film. The scan shows sharp XPS and Auger lines due to the main constituents (Ce, Sn, and O), whose identification was based on published values [12].

**Figure 3.**
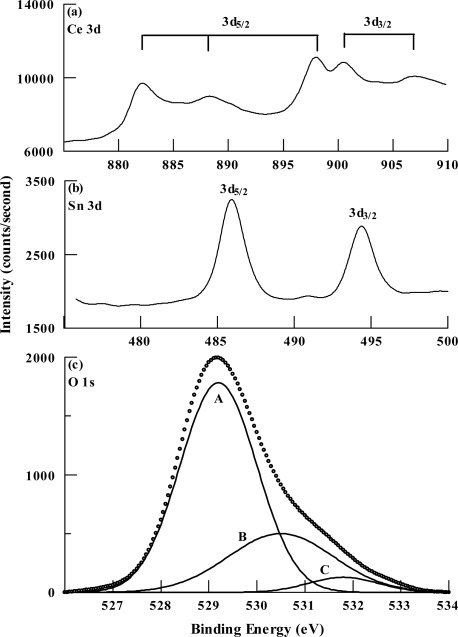
High resolution XPS spectra of the deposited films: (**a**) Ce 3d region: the assignment of the peaks to the two sublevels of Ce 3d is shown, (**b**) Sn 3d region, (**c**) O 1s region: the spectrum is deconvoluted into three components and the experimental spectrum is represented by the circles.

Detailed spectra in the Ce 3d, Sn 3d, and O 1s core level regions are shown in Figure 3. CeO_2_ has a relatively complex Ce 3d XPS spectrum that consists of six peaks, which correspond to the three pairs of spin-orbit doublets of oxidized CeO_2_ [13]. The measured Ce 3d spectrum (Figure 3(a)) clearly shows five peaks at binding energies of 882.4 eV, 888.1 eV, 897.8 eV, 900.4 eV, and 906.7 eV. The first three peaks correspond to Ce 3d_5/2_ and the last two peaks correspond to Ce 3d_3/2_. An additional peak at a binding energy of 916.6 eV was observed in the survey scan (shown by the arrow in Figure 2).

The binding energies of these peaks match very closely the reported values of the Ce 3d six peaks of cerium in the Ce^4+^ oxidation state [14]. These results indicate that cerium was present in the films as CeO_2_. This is further supported by the presence of the peak at 916.6 eV, which is considered as a fingerprint of the CeO_2_ phase [15]. The Sn 3d spectrum is shown in Figure 3(b). The spectrum shows the Sn 3d_5/2_ and Sn 3d_3/2_ peaks at binding energies of 485.9 eV and 494.4 eV, respectively. The reported values for the binding energies of the Sn 3d_5/2_ level in as-deposited tin oxide films were 486.6 in Sn^4+^ (SnO_2_) and 485.9 eV in Sn^2+^ (SnO) [16,17]. However, upon annealing in an oxygen atmosphere at 527 °C, tin oxide films became stoichiometric (SnO_2_) with a binding energy of the Sn 3d_5/2_ level at 486.1 eV (*i.e.*, a 0.5 eV down shift) [16]. Based on these results, we can assume that the tin was mainly present in our films as substoichiometric SnO_2_. The atomic concentration of the elements was calculated from the normalized areas of the peaks, taking the atomic sensitivity factors into account. The accuracy of determining this ratio is 10 %. The atomic concentrations were 0.20 for cerium, 0.13 for tin, and 0.67 for oxygen. The O1s spectrum (Figure 3(c)) was deconvoluted into three components using a Gaussian/Lorenzian mixed function employing Shirley background correction. The lower-energy component (A) with a binding energy of 529.1 eV corresponds to the Ce-O bond in CeO_2_ [16]. The medium-energy component (B) with a binding energy of 530.5 eV corresponds to the Sn-O bond in SnO_2_ [16,17]. This component provides further support for the conclusion that tin was mainly present as SnO_2_. The high-energy component (C) with a binding energy of 531.8 eV corresponds to oxygen atoms chemisorbed at the surface [16,17]. The weight of each component is obtained by dividing the area of that component by the total area of the O 1s spectrum. The weight of component A was 72.3%, the weight of component B was 25.4%, and that of component C was 2.3%. This shows that the predominant constituent of the films was cerium oxide. The intensity of the C component is partly related to the amount of adsorbed water, which in turn is proportional to the porosity of the films.

### 3.2. Optical Properties

**Figure 4.**
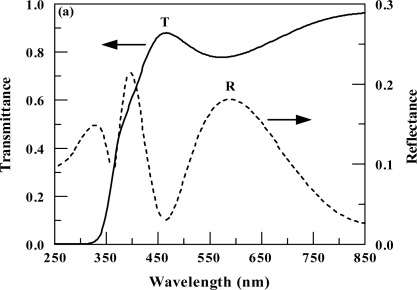
Normal-incidence transmittance (T) and reflectance (R) spectra of a deposited film of thickness 220 nm.

The normal-incidence reflectance and transmittance spectra, in the wavelength range *λ* = 250 – 850 nm, are shown in Figure 4. The transmittance spectra are obtained by dividing the measured transmittance by that of a fused silica substrate. The optical properties of the films can be used to estimate their porosity. The columnar microstructure of the films indicates the presence of voids within the films, which results in the films being porous. These voids (pores) may be filled with moisture (water) or air. The average packing density (*p*) is defined as the volume occupied by the solid part divided by the total volume occupied by the solid and voids. In the transparent region (*λ* > 500 nm), the refractive index of a film (*n_f_*) can be estimated from the minima in the transmittance spectra. The relation between the refractive index of the film (*n_f_*) and the packing density is given by [18]:

(1) $$n_{f}^{2}=\frac{(1-p)n_{v}^{4}+(1+p)n_{v}^{2}\;n_{s}^{2}}{(1+p)n_{v}^{2}+(1-p)n_{s}^{2}}$$

where *n_s_* is the bulk refractive index of the solid material, and *n_v_* is the refractive index of the voids (*n_v_* = 1.33 if the voids are filled with water or 1.0 if they are filled with air). The refractive index of the mixture film (n_f_ = 2.012) was obtained from the minimum in the transmittance (at λ = 572 nm) using the equation for the transmittance of a thin film on a transparent substrate [19]. The bulk refractive index of CeO_2_ and SnO_2_ is 2.5 and 2.0 respectively [20,21]. The bulk refractive index of CeO_2_ and SnO_2_ along with the XPS results were used to calculated the bulk refractive index of the mixture and found to be 2.305. Using these values in Equation (1), the packing density of the mixture was found to be 0.783 (for water-filled pores) or 0.884 (for air-filled pores). The porosity (*P*) is defined as: *P* = 1 – *p*. Thus, the porosity was in the range 11.6–21.7%. While porosity for pure CeO_2_ [8] reported earlier was 32%.

### 3.3. Atomic Force Microscopy

**Figure 5.**
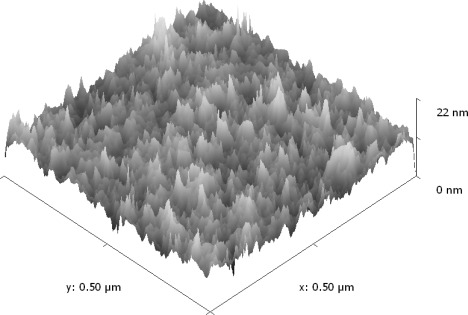
Shows tapping mode AFM images of SnO_2_ doped CeO_2_ thin film. The film thickness was 220 nm and annealed at 500 °C.

Figure 5 depicts the tapping mode AFM image of SnO_2_ mixed CeO_2_ thin film of thickness 220 nm, annealed at a temperature of 500 °C. The image reveals well separated conical nano columnar structure. This image has a measured roughness of (Area Ra 1.85 nm) and (Area RMS 2.5 nm), which shows that SnO_2_ mixed CeO_2_ films are highly porous with large surface area. This observation of high porosity confirms the similar findings of XPS and optical results discussed in Sections 3.1 and 3.2. The measured roughness reported earlier for pure CeO_2_ [10] was (Area Ra 2.38) and (Area RMS 3.11).

### 3.4. CO Gas-Sensing Properties

**Figure 6.**
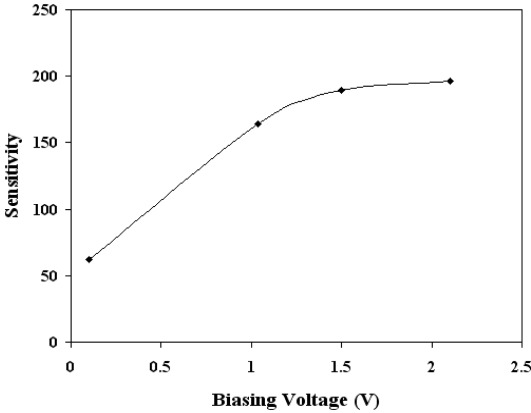
Sensitivity as a function of biasing voltage for SnO_2_ mixed CeO_2_ sensor of thickness 220 nm, CO concentration of 500 ppm and temperature of 430 °C.

Interaction of gaseous species with a thin film includes two steps [22]. First, oxygen from the ambient adsorbs on the surface of the film, and extracts electrons from the material, ionizes to O_2_^−^, O^−^ or O^2−^ depending on the operating temperature of the sensor (*T_op_*) [23–25]. The second step involves the reaction of the tested gas (CO in present case) with the adsorbed oxygen species. The introduction of a reducing gas (such as CO) decreases the resistance of the sensing film. For reducing gases, the sensitivity *S* is defined as (*ΔR/R_CO_*) × 100 where *ΔR* = *(R_air_* − *R_CO_)* [4], where *R*_air_ is the resistance of the film in air, and *R*_CO_ is the resistance of the film in the presence of CO. The sensitivity was measured as a function of sensor temperature and biasing voltages for different CO concentrations. Figure 6 shows the dependence of the sensitivity on the sensor bias voltage for SnO_2_ mixed CeO_2_ sensor at the optimum temperature of 430 °C, with a CO concentration of 500 ppm. Figure 6 depicts that there was significant variation in response for SnO_2_ mixed CeO_2_ sensor as the biasing voltages were increased.

**Figure 7.**
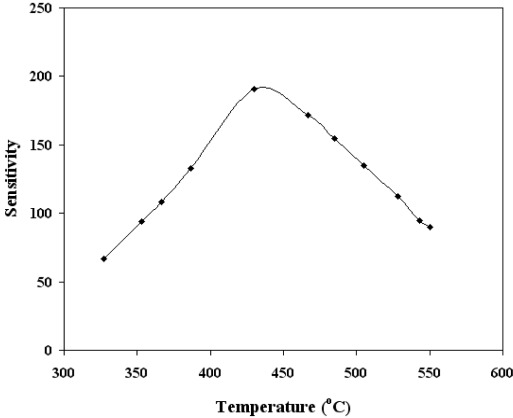
Sensitivity as a function of temperature for SnO_2_ mixed CeO_2_. The CO concentrations and films thickness was 500 ppm and 220 nm respectively.

**Figure 8.**
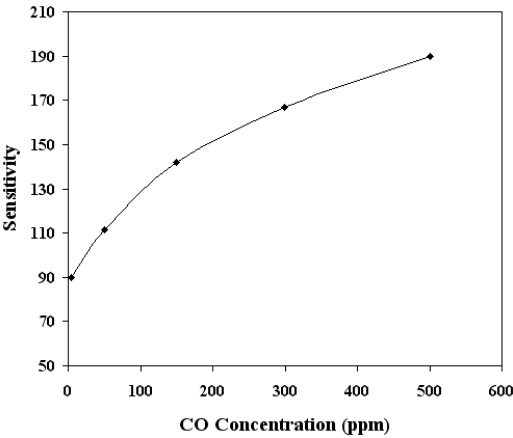
Response of the SnO_2_ mixed CeO_2_ thin film sensor to different CO concentrations. The film thickness was 220 nm and temperature of 430 °C.

Similar results were observed for other temperatures in the range 300–500 °C. Therefore, from these findings, the bias voltage was fixed at 1.5 V for subsequent experimental work. The effect of biasing voltages has been discussed in detail [10,26,27]. Briefly the effect of applied biasing voltage perhaps could be visualized as following; the applied biasing voltage would increase/decrease the Schottky barrier height (created by O^−^ adsorption), which in turn would increase/decrease the threshold CO concentration for the p-n or *vice versa* transitions. Figure 7 shows the sensitivity variation as a function of operating temperature (in the range 300 °C to 500 °C) for SnO_2_ mixed CeO_2_ film of thickness 220 nm and CO concentrations of 500 ppm. The inset of Figure 7 shows the peak value of the sensitivity for SnO_2_ mixed CeO_2_ is 430 °C. For the optimum operating temperatures 430 °C, the dependence of sensitivity on CO gas concentration was also investigated. The results are given in Figure 8, which shows that the sensor was capable of detecting CO gas concentrations as low as 5 ppm. It is clearly evident from Figures 7 and 8 that the SnO_2_ mixed CeO_2_ thin film gas sensor was highly sensitive to CO.

**Figure 9.**
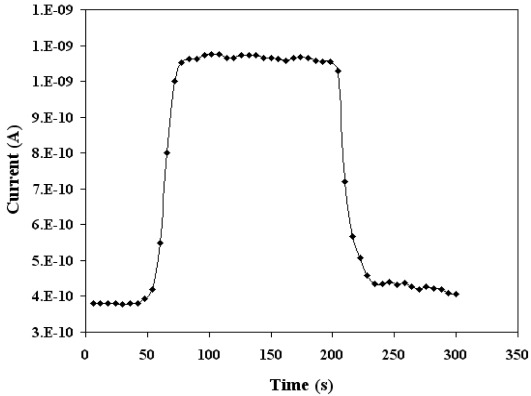
Current responses in time of SnO_2_ mixed CeO_2_ sensors exposed to 500 ppm of CO for a film thickness of 220 nm at the optimum temperature of 430 °C.

Figure 9 illustrates the dynamic current response of SnO_2_ mixed CeO_2_ sensor at the optimum temperature when exposed to 500 ppm of CO mixed in dry air. It is clear from the Figure 9 that for SnO_2_ mixed CeO_2_ sensor it took 26 seconds for the current to reach to its maximum value (peak response time). The sensors recover back to their initial value within 30 seconds. Comparing these results with the same sensing parameters for pure CeO_2_ [10], there is significant improvement in response time and detection limit, while the operating temperature has also increased for SnO_2_ mixed CeO_2_ sensors.

## 4. Discussion

The gas sensing parameters of SnO_2_ mixed CeO_2_ can be compared with those of the pure CeO_2_ films [8]. In the present case SnO_2_ mixed CeO_2_ had an optimum operating temperature that was higher (by about 40 °C) than that of pure CeO_2_. The response time for the SnO_2_ mixed CeO_2_ was much shorter than the pure CeO_2_, while the sensor recovery time for the two sensors were approximately the same. Also, there were significant differences in the CO gas detection limits. The SnO_2_ mixed CeO_2_ sensor was capable of detecting as small as 5 ppm of CO easily comparing with pure CeO_2_ sensors [10]. Although for pure CeO_2_ both surface roughness and porosity is higher than SnO_2_ mixed CeO_2_ and higher porosity and surface roughness enhances film sensitivity. However another important factor of acid-base reaction may have contributed in the improvement of sensing parameters for SnO_2_ mixed CeO_2_. The gas sensitivity in addition to porosity and surface roughness has also been related to the reactivity of the gas molecules over the sensing surface as determined by the acid-base character of the sensing surface. It was found that the basicity of the surface decreases as its electronegativity (χ) increases [28,29]. Increasing basicity increases the adsorption of the reducing gases such as CO. Thus decrease of the basicity of the surface leads to a reduction of the conversion efficiency of reducing gases such as CO [28,29]. Therefore the improved sensing parameters of SnO_2_ mixed CeO_2_ oxide could be due to the basicity of SnO_2_ mixed CeO_2_ which is much higher than the basicity of pure CeO_2_ [30]. Thus sensitivity in general depends on combination of several parameters discussed above; however it is possible that in specific cases one parameter may dominant the other.

## 5. Conclusions

Mixed metal-oxide thin films of tin oxide mixed cerium oxide were prepared by co-evaporation and were subsequently annealed in air at 500 °C for two hours. The thickness of the films was 220 nm. The films were amorphous with a uniform nano columnar structure. XPS measurements revealed that the films consisted of SnO_2_ and CeO_2._ The atomic concentration of the elements was calculated from the normalized areas of the peaks, taking the atomic sensitivity factors into account. The accuracy of determining this ratio is 10%. The atomic concentrations were 20% for cerium, 13% for tin, and 67% for oxygen. The XPS O 1s peak consisted of components arising from CeO_2_ and SnO_2_, as well as a component arising from adsorbed oxygen species. The presence of this component is an indication of the porosity of the films. Porosity was calculated using optical techniques, and was found to be in the range of 11.6–21.7%. AFM results indicated that SnO_2_ mixed CeO_2_ thin films exhibit large nicely separated conical nano columnar structure with the measured roughness of (Area RMS 2.5 nm). Such rough and porous films with increased basicity are highly suitable for gas sensing applications. The CO gas sensing properties of the SnO_2_ mixed CeO_2_ films were investigated. The optimum operating temperature, for a CO concentration of 500 ppm, was 430 °C. For this optimum temperature, the sensitivity of the films was found to be proportional to the CO concentration, and saturated for CO concentration higher than 5,000 ppm. The films were capable of detecting CO concentrations as low as 5 ppm. The response and recovery times were found to be 26 s and 30 s respectively. Such times are considered to be adequately fast for gas-sensing applications.
